# *Ligilactobacillus agilis* LA-V4 Isolated from Vulture Fecal Isolate: A Novel Probiotic Candidate with Broad-Spectrum Antibacterial Activity

**DOI:** 10.3390/pathogens15020148

**Published:** 2026-01-30

**Authors:** Siyuan Li, Chuxian Quan, Muhammad Farhan Rahim, Ping Sha, Jing Chen, Wenbin Shao, Jiakui Li

**Affiliations:** 1 College of Veterinary Medicine, Huazhong Agricultural University, Wuhan 430070, China; lsyhzau@webmail.hzau.edu.cn (S.L.); qcx2000@webmail.hzau.edu.cn (C.Q.); farhan092@webmail.hzau.edu.cn (M.F.R.); 2 Hubei Shishou Milu National Nature Reserve Management Office, Shishou 434401, China; 15272352338@163.com; 3 Hubei Provincial Wildlife Rescue and Research and Development Center, Wuhan 430075, China15515613057@163.com (W.S.)

**Keywords:** vulture, *Ligilactobacillus agilis*, probiotics, antimicrobial activity, vulture gut microbiota, pathogen inhibition

## Abstract

Vultures are extraordinarily adapted to feed on carrion, providing them with a constant microbiologically hostile environment. This peculiar ecological position has influenced the evolution of their gut microbiota, potentially conferring its uncommon antimicrobial traits and resistance to stress. In this study, we report on the isolation and comprehensive characterization of a lactic acid bacterium strain, identified as *Ligilactobacillus agilis*, from vulture feces via 16S rRNA gene sequencing. This strain exhibited potent antagonistic activity against several clinically relevant bacterial pathogens, including *Salmonella enterica* Typhimurium (25.26 ± 0.26 mm), *Escherichia coli* (23.5 ± 0.88 mm), *Staphylococcus aureus* (23.1 ± 1.8 mm), and *Listeria monocytogenes* (24.88 ± 0.61 mm), as demonstrated by agar well diffusion assays. Remarkably, it also demonstrated considerable resilience in simulated gastrointestinal conditions, with survival rates of 52.5 ± 7.4% in artificial gastric juice and 61.1 ± 3.7% in intestinal fluids. Antimicrobial susceptibility profiling confirmed its sensitivity to a broad range of commonly used antibiotics, including gentamicin, streptomycin, clindamycin, and penicillin. Whole-genome sequencing further revealed a complete repertoire of core genes associated with genetic information processing, robust carbohydrate metabolism, and nutrient assimilation, underscoring its adaptability and probiotic potential. It is important to note that the analysis of the assembled genome against VFDB did not show the presence of any known virulence factor according to the given criteria, which is preliminary evidence of safety-related aspects that are to be followed with the help of guideline-based analyses. Taken together, the unique ecological origin and in vitro inhibitory activity against the tested pathogens, gastrointestinal robustness, genomic features, and safety credentials position this *L. agilis* strain as a promising probiotic candidate for mitigating enteric infections in animal production systems, warranting further functional validation and in vivo efficacy studies.

## 1. Introduction

The World Health Organization (WHO) has emphasized that antimicrobial resistance (AMR) constitutes one of the most pressing threats to the health and financial well-being of the global population [1]. The AMR refers to the ability of microorganisms, especially bacteria, to survive the action of antimicrobial agents by employing various resistance mechanisms. A primary driver of this escalating crisis is the inappropriate and unregulated use of antibiotics, with the agricultural sector being a key contributor [2,3]. Historically, antibiotics have been applied in intensive livestock production systems where they are used not only to treat, but to grow, as well as prevent diseases routinely in animals that are clinically healthy. These are, however, now prohibited or highly limited in a variety of areas (e.g., AMG growth promoters banned by the EU, and subsequent restrictions on non-therapeutic use), and use is increasingly regulated and veterinary indication-based; however, inappropriate or non-therapeutic use remains the cause of de-development and dissemination in AMR [4]. This indiscriminate practice accelerates the selection and enrichment of multidrug-resistant bacterial strains within the animal microbiome, which directly participate in zoonotic transmission cycles, and elevates the risks of treatment failure, mortality, morbidity, and hospitalization in the human population [5].

With increasing restrictions on the use of antibiotics in food production, stakeholders and scientific communities in the world are trying all ways to find solutions to disease prevention in farming through alternatives that are viable and sustainable [6]. Probiotics have received great interest amongst them as potential bio-interventions. Defined as live microorganisms that, when administered in sufficient amounts, confer health benefits to the host, probiotics have been shown to have the ability to regulate gut microbiota, increase nutrient absorption, bolster mucosal immunity, and prevent pathogen colonization [7,8].

Importantly, specific probiotic strains, most notably those within the Lactobacillus genus, have demonstrated protective activity against *Salmonella* spp., the leading pathogen responsible for over half of foodborne disease cases globally [9,10,11,12]. Probiotics supplementation around the early developmental stages has been linked with enhanced growth performance and infection resistance in poultry, most importantly with the help of competitive exclusion and immunomodulatory processes [13,14].

Notable among these beneficial microbes is the flagellated lactic acid bacterium *L. agilis*, which has emerged as a research focus in recent years due to its robust acidogenic potential and ability to modulate intestinal mucosal health. It has been previously documented that *L. agilis* and *Lactobacillus salivarius* can regulate the activity of intestinal stem cells through crypt niche signaling [15], have protective properties against *E*. *coli*-induced enteritis in mouse models, and suppress *Campylobacter* spp. in vitro [16,17]. Despite these findings, the majority of probiotic research continues to be confined to traditional paradigms, such as gut isolates and fermented food-derived strains, whereas microbial communities with high adaptability to extreme ecological habitats remain underexplored.

Vultures, being the sole obligate terrestrial vertebrate scavengers, have a decided ecological niche characterized by the frequent consumption of decomposing carcasses. This necrophagic type of food exposes them to extremely high concentrations of pathogenic microorganisms, enteric microorganisms, and zoonotic microorganisms. However, vultures can hardly be affected by site-related infections, which is why their intestinal microbiota is highly specialized and protective [18,19,20]. Metagenomic analyses of the vulture gastrointestinal tract, as a matter of fact, have demonstrated that the intestines of the vulture are capable of neutralizing or eradicating pathogenic bacteria that it obtains during the feeding process, presumably because of a co-evolved microbiome exhibiting an exceptional tolerance to stress and antimicrobial capabilities. Notwithstanding the strong ecological argument, studies on probiotic candidates found in the vulture gut are still very limited.

This is to report the isolation of a *L. agilis* strain of vulture-origin, named LA-V4, which was isolated using freshly voided fecal material collected non-invasively on healthy birds. We proceeded to strain-level phenotypic and genomic characterization to test its candidacy as a type of probiotic organism. LA-V4, in particular, was evaluated in antagonistic activity against a panel of major indicator pathogens, tolerance to simulated gastric and intestinal conditions, and resistance to clinically relevant antibiotics. Simultaneously, to facilitate taxonomic validation and genome-based screening of functional potential and safety-relevant phenotypes, such as the presence/absence of annotated virulence-associated determinants and resistance-associated genes, was identified by long-read whole-genome sequencing. All these are used together to create a standardized baseline datum of LA-V4 and provide a justification for further mechanistic and in vivo validation studies. Our findings demonstrate that this strain has exclusive probiotic potential and can be used down the line to help in countering bacterial infections in food animals taken under antibiotic-cut or antibiotic-free conditions.

## 2. Materials and Methods

### 2.1. Isolation and Identification

Fresh fecal samples were obtained in a non-invasive manner in clinically healthy vultures. To reduce selection bias and standardize the criteria of inclusion, freshly voided feces were only sampled, and material with apparent environmental contamination (soil or debris) was avoided. Samples were aseptically transferred into sterile containers, immediately put under ice-cooled conditions, and taken to the laboratory to undergo processing within 3 h after collection. Fecal material was suspended and homogenized under aseptic conditions in sterile phosphate-buffered saline (PBS) upon arrival, then left to settle by gravity, and the resulting supernatant was plated (100 μL) on de Man, Rogosa, and Sharpe (MRS) agar using the spread-plate technique. Plates were incubated at an aerobic temperature of 37 °C for 24–48 h. Three consecutive streaks on fresh MRS agar were used to isolate morphologically distinct colonies, which were identified and purified to give single-colony isolates, which were further grown in MRS broth at 37 °C over 24 h. Gram staining was performed to perform preliminary characterization. Taxonomic identification was performed by 16S rRNA gene sequencing: genomic DNA was isolated through the Bacterial Genomic DNA Extraction Kit (Vazyme Biotech, Nanjing, China), the 16S rRNA gene was amplified with universal bacterial primers, and the obtained sequences were assigned to the species level by matching them with the reference database.

### 2.2. Growth Time Curve of Isolates

All the bacterial isolates were inoculated in de Man, Rogosa, and Sharpe (MRS) broth at an inoculum ratio of 1% (*v*/*v*) and allowed to incubate at 37 °C in a shaking incubator, which was to this effect adjusted to 180 RPM. Bacterial growth was monitored by measuring OD 600 at 2 h intervals for 24 h. Each time point was recorded in triplicate.

### 2.3. Assay of the Antimicrobial Activity

The antimicrobial activity of LA-V4 against *Salmonella enterica* Typhimurium (clinical isolate), *Staphylococcus aureus* (ATCC 25923), *Escherichia coli* (ATCC 25922), and *Listeria monocytogenes* (clinical isolate) was determined using the Oxford cup diffusion method [21]. The detailed experimental procedures were as follows: First, each indicator strain was individually inoculated into LB broth medium and cultured with constant temperature shaking at 37 °C and 180 r/min for 12–16 h. The indicator bacterial suspension was then adjusted to a concentration of approximately 10^6^ CFU/mL and stored as the indicator working suspension for subsequent use. LA-V4 was inoculated into MRS broth medium and cultured with constant temperature shaking at 37 °C and 180 r/min for 24 h to obtain the probiotic fermentation broth. The fermentation broth was centrifuged at 2000 RPM for 10 min to remove the precipitated bacterial cells, and the supernatant was collected. Subsequently, the pH of the collected supernatant was adjusted to 6.5–7.0 using 1 mol/L sodium hydroxide solution. The aforementioned indicator working suspension was uniformly spread on the surface of LB agar plates. Sterile Oxford cups were aseptically placed on the surface of the indicator-inoculated agar plates, and 150 μL of the prepared neutral 150 μL of the prepared neutralized, cell-depleted fermentation supernatant was accurately added into each Oxford cup. Finally, incubation and antimicrobial activity determination were performed: the inoculated agar plates were incubated in a constant temperature incubator at 37 °C for 12–16 h. Indicator cultures were adjusted to approximately 1 × 10^6^ CFU/mL by serial dilution based on optical density (OD) and were verified by plate counting. After incubation, the diameter of the inhibition zone around each Oxford cup was measured using a vernier caliper (unit: mm). Each treatment was conducted in three parallel replicates, and the average value was taken as the final result. Since neutralized, cell-free supernatant only was tested, and no pH-matched medium or enzyme-treated controls were used, this assay was intended to record inhibition by the neutralized supernatant and not to identify the underlying antimicrobial effect.

### 2.4. Tolerance of Isolates to Artificial Gastric Intestinal Fluids

MRS broth (37 °C, 180 rpm) was used to prepare the overnight cultures of LA-V4. Cells were inoculated (1% *v*/*v*) into simulated gastric fluid (SGF) and simulated intestinal fluid (SIF) and allowed to incubate at 37 °C over a period of 3 h. The growth control was MRS broth in the absence of simulated fluids. Plate counting was used to determine viable counts (CFU/mL) before and after exposure, and the rate of survival was calculated as follows. The pharmacopeial test-solution formulations have been used to define SGF and SIF to facilitate reproducibility. SGF was made of a mixture of sodium chloride (2.0 g/L) and pepsin (3.2 g/L) in water that were acidified with hydrochloric acid (7.0 mL/L) to achieve a pH of 1.2. SIF was composed of potassium phosphate monobasic (6.8 g/L) and sodium hydroxide solution (0.2 M; 190 mL/L) followed by the addition of pancreatin (10.0 g/L), and pH was brought to 7.5 ± 0.1. Both of these formulations were freshly added with enzymes. To be more precise, USP-type SGF/SIF are very common simplified standardized models; re-reported osmolarities are of the order of 180 mOsmol/kg of SGF and 110–114 mOsmol/kg of SIF buffers, depending on the precise formulation.

(1) $$Survival\;rate\left(\%\right)=\frac{NO.\;\;of\;colonies\;after\;treated}{NO.\;\;of\;colonies\;in\;contral\;group}\times 100\%$$

### 2.5. Hemolytic Activity

The hemolytic activity was assessed using a standard blood agar plate assay. Bacterial suspensions of the test strain *Ligilactobacillus agilis* LA-V4 and the hemolytic positive control strain *Staphylococcus aureus* ATCC 25923 were prepared aseptically. Single colonies were picked with a sterile inoculating loop and streaked or spread onto Columbia blood agar plates containing 5% sterile defibrinated sheep blood. The plates were then incubated at 37 °C for 24 h. After incubation, hemolytic patterns were examined against a white background. β-Hemolysis was identified by a clear, transparent zone completely surrounding the colony. α-Hemolysis was characterized by a partial hemolysis resulting in a greenish or brownish-green discoloration around the colony. The absence of a visible hemolytic zone was classified as γ-hemolysis (non-hemolytic). In this assay, *Staphylococcus aureus* ATCC 25923 served as the positive control and consistently produced a characteristic β-hemolytic zone, thereby validating the efficacy of the medium and the experimental system. The hemolytic phenotype of the test strain LA-V4 was determined by direct comparison with this control.

### 2.6. Biochemical Identification

The strain selected was based on the exploitation of conventional biochemical test tubes according to the fulfilling processes in Bergey’s Manual of Determinative Bacteriology. The fermentation profiles of the carbohydrate strains were identified based on the ability of the strain to ferment a range of sugars. Other biochemical characteristics, like gelatin liquefaction and hydrogen sulfide (H_2_S) production, were also investigated to assist in phenotypic identification.

### 2.7. Antibiotic Susceptibility of Isolates

According to Charteris et al., the disk diffusion method was used to determine the antibiotic susceptibility of the isolates [22]. The lone isolate was incubated in MRS broth to a concentration of 1 × 10^8^ CFU/mL. Following thorough mixing, 100 µL was transferred to MRS agar medium, with the fresh bacterial suspension being evenly distributed on it. After some antibiotic disks were applied on agar surface, the plates were incubated anaerobically at a temperature of 37 °C for 24 h. Then the measurement of the diameters of the circles of inhibition was performed.

### 2.8. General Genome Features of LA-V4

*Ligilactobacillus agilis* LA-V4 was cultured for 16–18 h in the MRS broth at 37 °C. After incubation, the centrifugation of bacterial cells was performed at 5000 rpm for 2 min and followed by washing using sterile phosphate-buffered saline (PBS), three times. The sodium dodecyl sulfate (SDS)–based lysis system coupled with commercial purification columns was used to extract genomic DNA with high purity and yield. The nucleic acid extraction and sequencing were performed by BIOYIGENE (Wuhan, China). The purity of the DNA was determined by a NanoDrop ONE spectrophotometer (NanoDrop Technologies, Wilmington, DE, USA) on the basis of A260/A280 ratios, and the concentration of the DNA was calculated using a Qubit 3.0 fluorometer (Thermo Fisher Scientific, Waltham, MA, USA). Whole-genome sequencing was conducted on the Promethion platform (Oxford Nanopore Technologies, Oxford, UK), which offers long-read high-throughput sequencing data to examine the genome in detail. After removing low-quality reads and short fragments (length < 2000 bp) from the raw sequencing data, the cleaned data were subjected to assembly analysis. Hybrid assembly of the quality-controlled data was conducted using Unicycler 0.5.1, and the assembled sequences were functionally annotated with InterProScan. Genome-encoded proteins were extracted and annotated by sequence alignment against multiple databases: GO (Gene Ontology), COG (Clusters of Orthologous Groups), CARD (Comprehensive Antibiotic Resistance Database), VFDB (Virulence Factor Database), CAZy (Carbohydrate-Active enZymes database), and PHI (Pathogen–Host Interactions database) using bioinformatics tools including BLASTp, ABRicate, and InterProScan.

### 2.9. Statistical Analysis

Parallel replicates of independent cultured cultures were used to carry out all quantitative experiments. Continuous variables including inhibition-zone diameters, optical density at 600 nm (OD600), and survivability in simulated gastric and intestinal fluids were measured and are reported as mean ± standard deviation (SD). The number of experimental replicates is stated.Since the study was founded on one isolate and intended to determine the functional character of the strain level, as opposed to population level inference, no association modeling (e.g., genotype-phenotype linkage across multiple strains), temporal trend analyses, or clinical correlation analyses were performed. The statistical analysis was consequently aimed at reproducible estimation of the effect size and variability between replicate assays.

## 3. Result

### 3.1. Isolation and Identification of Ligilactobacillus agilis

**Figure 1:**
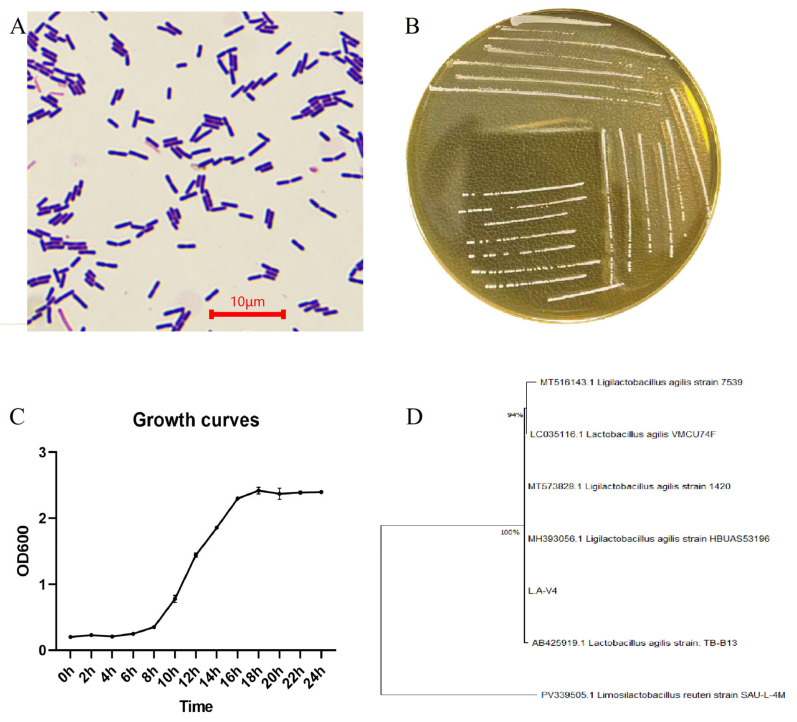
(**A**) Gram staining morphology; (**B**) colony morphology; (**C**) growth curve of LA-V4 in MRS broth; (**D**) phylogenetic tree of isolated strains.

The isolate grew smooth, circular, cream/milky-white colonies on MRS agar with a uniform margin (Figure 1B). Gram staining and microscopic analysis revealed Gram-positive, short, rod-shaped cells, which appeared individually and in small chains with an approximate cell length within the micrometer range (usually 2–5 μm under the conditions examined; Figure 1A). Comparison of 16S rRNA gene amplification and sequencing, followed by BLAST similarity searching and constructing a phylogenetic tree, assigned the isolate to the *L. agilis* clade; the strain was consequently renamed as *L. agilis* LA-V4 (Figure 1D). Growth-curve analysis of MRS broth showed that in the initial 8 h of culture, the bacteria were in the lag phase, followed by a rapid growth with 8–16 h, and a subsequent transition into a stationary phase, as illustrated in Figure 1C, with OD 600 leveling off at 18–24 h under the maintenance conditions.

### 3.2. Antimicrobial Activity of the L.A-V4

**Figure 2:**
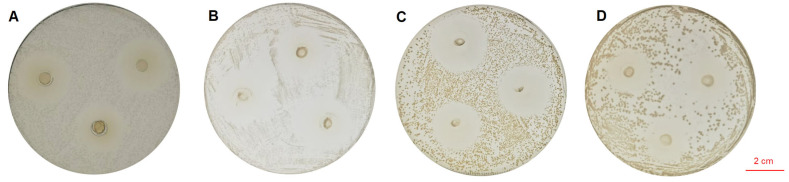
(**A**) In vitro inhibitory activity of LA-V4 against *S. typhimurium*; (**B**) in vitro inhibitory activity of LA-V4 against *E. coli*; (**C**) in vitro inhibitory activity of LA-V4 against *S. aureus*; (**D**) in vitro inhibitory activity of LA-V4 against *L. monocytogenes*.

The cell-free fermentation supernatant of LA-V4 (pH 6.5–7.0) was neutralized to obtain a clear inhibition zone against the tested indicator pathogens with a diameter of 25.26 ± 0.26 mm (*S. typhimurium*), 23.50 ± 0.88 mm (*E. coli*), 23.10 ± 1.80 mm (*S. aureus*), and 24.88 ± 0.61 mm (*L. monocytogenes*) (Figure 2).

### 3.3. Gastrointestinal Fluid Tolerance, Hemolytic Activity, and Biochemical Identification of LA-V4

**Figure 3:**
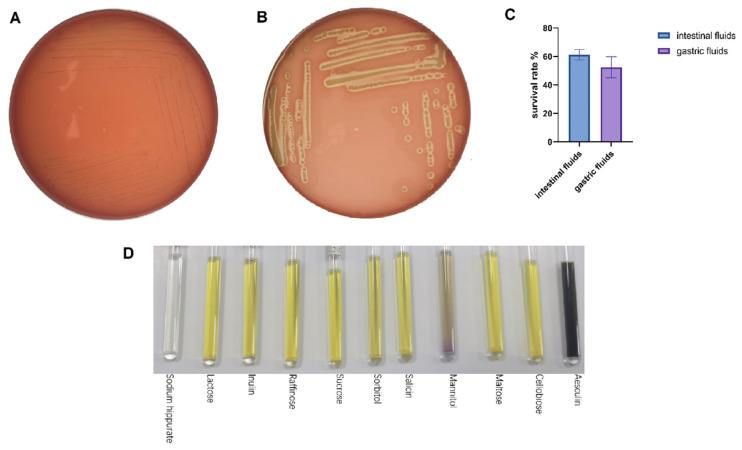
(**A**,**B**) Negative hemolytic activity of LA-V4 and positive control of *S. aureus* (5% sheep blood agar); (**C**) gastric fluid survival rate and intestinal fluid survival rate; (**D**) the biochemical test result of isolate LA-V4.

The survival during digestive transit was tested with artificial gastric and intestinal fluids, which is a widely used in vitro surrogate endpoint to determine tolerance to simulated gastrointestinal conditions [23]. The survival during digestive transit was tested with artificial gastric and intestinal fluids, which are widely used in vitro surrogate endpoints to determine tolerance to simulated gastrointestinal conditions [22]. LA-V4 survived the two challenges with a survival rate of 52.5 ± 7.4 in artificial gastric juice and 61.1 ± 3.7 in artificial intestinal juice (Figure 3C). Blood agar screening on safety revealed distinct β-hemolysis surrounding the *S. aureus* positive control and γ-hemolysis in LA-V4 (no hemolytic reaction detected) and no clearing zone around colonies (Figure 3A,B). In addition, micro-biochemical profiling revealed a generalized pattern of carbohydrate use. LA-V4 fermented/assimilated several carbon sources, such as lactose, inulin, raffinose, sucrose, sorbitol, salicin, mannitol, maltose, cellobiose, and esculin (Figure 3D; Table 1), and failed to hydrolyze sodium hippurate (Table 1).

### 3.4. Antibiotic Susceptibility of LA-V4

The antibiotic susceptibility profile indicated that LA-V4 was sensitive to a number of antimicrobial agents (Table 2), including gentamicin, streptomycin, clindamycin, and penicillin, indicating a favorable safety profile for potential probiotic application (diameter > 25 mm); moderately resistance to oxacillin and ciprofloxacin (15 mm < d < 19 mm); and high resistance to erythromycin, norfloxacin, and kanamycin (d < 15 mm).

### 3.5. Genomic Characterization of Ligilactobacillus agilis LA-V4

**Figure 4:**
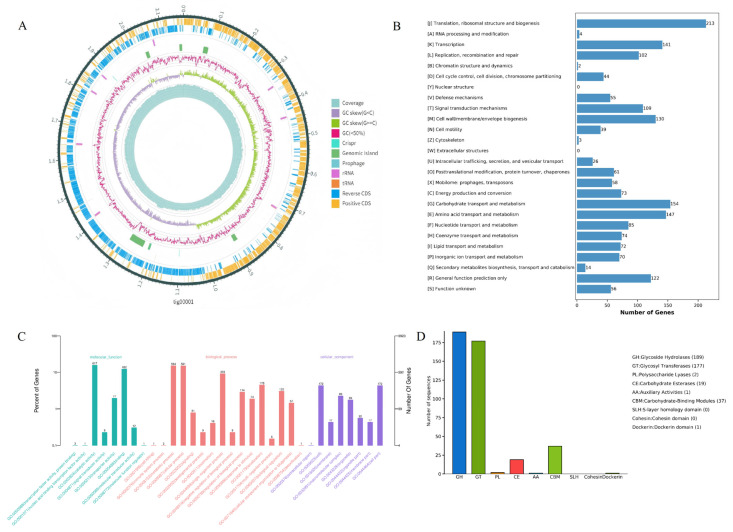
(**A**) Circular genome map of LA-V4; (**B**) COG functional classification statistics of proteins encoded by the LA-V4 genome; (**C**) GO functional classification statistics of proteins encoded by the LA-V4 genome; (**D**) CAZy functional classification statistics of proteins encoded by the LA-V4 genome.

The complete genome of LA-V4 consists of five contigs with a total length of 2,203,196 bp(Figure 4A), including one chromosomal gene and four plasmids (one of which is a linear plasmid). Gene prediction results revealed that the genome encodes 2070 complete coding sequences (CDSs). Through Gene Ontology (GO) functional classification annotation, 1211 genes (accounting for 58.50% of the total CDSs) were effectively annotated (Figure 2A), and these annotated genes can be further categorized into three core functional groups: molecular function, biological process, and cellular component. The results of the Cluster of Orthologous Groups (COG) database annotation showed that a total of 1628 genes in the LA-V4 genome were annotated, accounting for 78.65% of the total CDSs, and these genes were classified into 26 COG functional categories (Figure 4B). Among them, the top eight categories with the highest number of enriched genes were as follows: translation, ribosomal structure, and biogenesis (213 genes), carbohydrate transport and metabolism (154 genes), amino acid transport and metabolism (147 genes), transcription (141 genes), cell wall/membrane/envelope biogenesis (130 genes), general function prediction only (122 genes), signal transduction mechanisms (109 genes), and replication, recombination, and repair (102 genes). In addition, a certain number of genes were enriched in coenzyme transport and metabolism (74 genes) and lipid transport and metabolism (72 genes) (Figure 4C). These results suggest that LA-V4 may play a role in the intestinal microecological environment by participating in the transport and metabolism of various nutrients (e.g., carbohydrates, amino acids, and lipids) and energy metabolism processes in the host intestine. BLAST analysis against the Virulence Factor Database (VFDB) revealed that LA-V4 does not harbor any virulence genes. Genome annotation to CARD has found four potential antimicrobial resistance determinants (*erm*C, *tet*M, *tet*(L), and *poxt*A). During phenotypic testing, the organism (LA-V4) was vulnerable to doxycycline and linezolid under the conditions (Table 2). This phenotype–genotype mismatch suggests that annotated AMR genes are not sufficient evidence of functional resistance in our test conditions (e.g., the genes are incomplete, not expressed, or provide resistance only in particular conditions). Thus, such CARD findings are presented as genomic annotations, which need subsequent characterization, such as the assessment of gene integrity, expression potential, and genetic context. [24,25]. The antibiotic susceptibility test showed no resistance of LA-V4 to doxycycline (d > 28 mm). In the CAZymes database annotation, a total of 375 Carbohydrate-Active enZyme (CAZyme)–related genes were identified in the LA-V4 genome. Most of these genes were classified into the Glycoside Hydrolases (GHs, 189 genes) and Glycosyl Transferases (GTs, 177 genes) families; additionally, 37 genes were annotated to Carbohydrate-Binding Modules (CBMs) and 19 genes to Carbohydrate Esterases (CEs) functional categories (Figure 4D). The annotation of a large repertoire of CAZyme-related genes suggests that LA-V4 has broad carbohydrate metabolic potential, which may support adaptation to carbohydrate-variable intestinal environments. However, no host-specific adhesion or in vivo colonization experiments were performed in this study, and therefore, colonization in poultry cannot be inferred from the genomic data alone.

## 4. Discussion

Vultures routinely ingest carcasses that contain dense and diverse microbial loads, including enteric pathogens and spoilage-associated bacteria. It is also indicative of the fact that the host physiology and resident gut microbiota might be working together to provide excellent defenses against microbial invasion and toxin-mediated damage, since these birds tend to be healthy despite unceasing exposure. In this ecological sense, the vulture’s gastrointestinal tract is a largely untapped niche of bacteria that could have developed increased stress resistance and antagonistic behavior toward other microorganisms. In our current study, we selected a lactic acid bacterium from the feces of vultures and named the bacterium *L. agilis* (LA-V4) using 16S rRNA sequencing and phylogenetic evaluation. We also prove the identity that LA-V4 exhibits wide-spectrum inhibition of numerous medically and agronomically significant pathogens, is stable at simulated gastrointestinal environments, is non-hemolytic, and possesses genomic characteristics of metabolic plasticity.

All these findings taken together argue towards the concept that vulture-related lactic acid bacteria research can be utilized as some promising probiotics in animal production systems [26]. The current study was in line with this requirement by assessing a lactate-derived *L. agilis* isolate (LA-V4) as a potential probiotic of relevance with wide antagonistic effects against enteric and opportunistic animal isolate pathogenicity. The ecological conceptual strength of this work is the rationale provided for why vultures serve as the source of probiotic candidates, since they constitute an extreme dietary niche with regular contact with high loads of microbes due to carrion [27]. Vultures also exhibit physiological barriers that shape and constrain microbial survival in the gastrointestinal tract, most notably a highly acidic stomach environment that can reach pH values near 1.0 in field measurements. Consistent with this idea, metagenomic work in New World vultures has described a highly specialized gut microbiome and evidence of strong gastrointestinal selectivity, supporting the broader hypothesis that vulture-associated microbial communities may contribute to colonization resistance against pathogens. As the only obligate necrophages among terrestrial vertebrates [28], vultures are chronically exposed to various pathogenic microorganisms that cause diseases such as anthrax, tuberculosis, and brucellosis [29]. The isolate characterized here was identified by 16S rRNA sequencing as *Lactobacillus agilis*; however, it is worth noting that the former genus *Lactobacillus* has been taxonomically reorganized, and *L. agilis* is now commonly placed within the genus *Ligilactobacillus* (i.e., *Ligilactobacillus agilis*) in updated classifications [30].

In this study, *Ligilactobacillus agilis* LA-V4, isolated from vulture feces, showed several in vitro properties that are commonly used as preliminary indicators of probiotic candidacy. LA-V4 produced clear inhibition zones against both Gram-negative and Gram-positive indicator organisms in the Oxford cup assay, and it maintained viability under simulated gastrointestinal challenges. In addition, the strain was non-hemolytic on blood agar. Together with the genome-based annotation indicating broad carbohydrate metabolic capacity, these findings suggest that LA-V4 may have probiotic potential, but they should be interpreted as strain-level in vitro evidence rather than confirmation of efficacy in a target host [16,17]. LA-V4 displayed strong in vitro inhibition of both Gram-negative and Gram-positive indicator organisms, including *S*. *typhimurium*, *E. coli*, *S. aureus*, and *L. monocytogenes* (reported here as inhibition-zone diameters in the Oxford cup assay). Broad-spectrum inhibition of this type is consistent with the well-documented capacity of *Lactobacilli* to suppress pathogens through multiple, often synergistic mechanisms rather than a single bactericidal factor, including the production of organic acids, bacteriocins, and competition for nutrients [31,32,33,34,35]. A previous study confirmed that *L. agilis* exhibits significant in vitro inhibitory activity against *E. coli*. This antibacterial effect suggests that LA-V4 may have potential to antagonize intestinal pathogenic microorganisms and support the prevention of intestinal infections. Previous studies have indicated that lactic acid bacteria can inhibit the colonization and proliferation of competing bacteria through multiple mechanisms. Beyond antibacterial activity, accumulating experimental evidence has explicitly validated the biological activities of *L. agilis* in antagonizing inflammatory responses and alleviating oxidative damage [16,36], further supporting its probiotic value. Secondly, LA-V4 showed remarkable tolerance to artificial gastrointestinal juices, which is a critical prerequisite for its successful colonization and survival in the host’s gastrointestinal tract [37]. Tolerance to the acidic environment of the stomach and bile salt stress in the intestine ensures that LA-V4 can reach the distal intestine in an active state, thereby laying a solid foundation for exerting subsequent probiotic functions.

Additionally, LA-V4 exhibited no hemolytic activity, indicating a high level of biosafety and eliminating the potential risk of hemolytic damage to the host. LA-V4 was capable of efficiently fermenting various carbon sources, including lactose, inulin, and raffinose, and this characteristic was highly consistent with the results of genome annotation. Efficient carbohydrate metabolism capacity is a core foundation for probiotics to colonize and exert their functions in the host [38,39,40]. This capacity enables LA-V4 to better adapt to the complex nutritional environment of the host intestine, facilitates its proliferation in competition with the indigenous intestinal flora, and thereby regulates the balance of the intestinal microbiota.

Whole-genome sequencing (long-read) and annotation guided by databases, and subsequent screening, such as the Virulence Factor Database (VFDB), failed to identify familiar virulence-associated determinants in strain LA-V4, which justifies a positive preliminary safety profile when limited by available reference materials. Genome annotation has also identified antimicrobial-resistance genes (*erm*C, *tet*M, *tet*(L), and *poxt*A); although phenotypic tests did not show any corresponding doxycycline-resistant phenotype in the tested conditions, this highlights the fact that the presence of genes does not always indicate functional resistance. These determinants were found in the assembled genome, not on plasmid contigs, but on the chromosomal sequence; however, the possibility of horizontal mobility cannot be determined based on an isolated isolate or genome, and must be investigated using specific mobile-element studies and experimental transferability experiments in future investigations [41,42,43].

Functionally, LA-V4 showed strong enrichment of carbohydrate transport/metabolism functions and a large repertoire of CAZyme-associated genes in the CAZy annotation, with many assigned to glycoside hydrolase (GH) and glycosyl transferase (GT) families (reported here as counts from this study). CAZymes are central to the microbial capacity to degrade, modify, or synthesize complex carbohydrates, and CAZy is the standard reference database used to classify these enzyme families and domains [42]. For LA-V4, expanded carbohydrate-active potential may plausibly support persistence in carbohydrate-variable intestinal conditions and could contribute indirectly to pathogen suppression through resource competition and production of inhibitory fermentation metabolites [35]. Notably, all four aforementioned resistance genes were located in the chromosomal genome of the strain rather than on transferable plasmids, suggesting a low potential risk of horizontal transmission of the resistance genes from LA-V4 [24,25,43].

Taken together, these findings reveal that *L. agilis* LA-V4 has quantifiable in vitro antagonistic activity with a number of indicator pathogens, withstands exposure to simulated gastric and intestinal fluids, and has an absent hemolytic phenotype under the conditions under which the experiment was conducted. Moreover, genome-based functional annotation and initial safety screening (including absence of annotated VFDB virulence determinants and the presence of resistance-associated genes, which should be followed up by further examination) is aided by long-read whole-genome sequencing. These results can best be regarded as strain-level evidence of probiotic candidacy and not evidence of efficacy, mechanism, or field performance. In particular, the current dataset has not defined the molecular basis of antagonism, confirmed colonization or persistence in vivo, or protection against infection in a target host.

We, therefore, make LA-V4 a candidate to be further developed, and the next steps with priority to follow include mechanistic dissection of inhibitory activity, more comparative tests against already known probiotic reference strains under standardized conditions, as well as in vivo tests to measure safety, colonization dynamics, and efficacy. Since this study focuses on one *L. agilis* isolate (LA-V4), this dataset is not in a format suitable to provide association modeling or temporal/clinical trends. Subsequently, the findings are viewed as strain-level functional readouts being replicate-based quantitative reports, as opposed to population-level statistical associations. In spite of the supportive in vitro phenotyping and genome-informed safety/functionality screening, the current research is limited by a number of limitations on interpretability and wider generalization. Notably, it is not a retrospective work that relies on the solitary population of a clinical center; however, an isolate-based characterization based on a specific ecological source. The major constraint of external validity is that conclusions are made based on a mono-strain (LA-V4) obtained by digestion in vulture feces, and strain-level heterogeneity by strain *L. agilis* and among populations of vultures is probable. As a result, we need to interpret our findings as evidence of candidate probiotic potential depending on the demonstrated antibacterial activity and genomic characteristics, but not necessarily as evidence that a consistent performance is observed in different hosts or environments. Future research directions are to scale up sampling to more subjects and locales, to compare LA-V4 with known probiotic reference strains in controlled environments, and to localize different inhibitory activities (e.g., organic acids, bacteriocin-like activity, competitive exclusion). Last, due to the existence of numerous plasmids and annotated antimicrobial resistance determinants, selecting mobility/transferability studies and in vivo validation (efficacy, colonization dynamics, host reactions, and safety) will be critical in advance of any recommended practical application.

## 5. Conclusions

*Ligilactobacillus agilis* LA-V4, a lactic acid bacterium isolated from the vulture’s liquid feces, was found to have in vitro antagonistic activity against major food and enteric pathogens, shown by sequencing of the 16S rRNA gene. The strain was able to inhibit *S. typhimurium*, *E. coli*, *S. aureus*, and *L. monocytogenes*, and a range of 23 to 25 mm was found in all varieties. This strain survived simulated gastrointestinal conditions (52.5% in artificial gastric juice; 61.1% in intestinal fluid) and showed susceptibility to several antibiotics in vitro; however, genome annotation identified putative AMR determinants, and additional work is required to determine their integrity, expression potential, and transferability before considering application. The potential of this bacterium as a probiotic was validated by whole-genome sequencing, which suggested that it had a metabolic capacity associated with anchoring to the intestines, and the VFDB screening did not find virulent factors. Its vulture-based creation provides an ecological niche, and its functionality highlights its possible applications in animal production systems as a probiotic alternative. Future research must aim at in vivo efficacy studies, a mechanistic study of its antimicrobial compounds, and the effect of its effect on host microbiota composition and immune responses. In short, *L. agilis* LA-V4 is a vulture-derived isolate, which exhibits extensive in vitro antagonistic activity against major enteric/foodborne pathogens, simulated gastric intestinal tolerance, no hemolytic phenotype, and genome-informed functional and safety screening (no annotation VFDB virulence determinants present). These findings argue in favor of LA-V4 as a potential area of development in the conditions of antibiotic-reduction measures in animal production, but also note that application-oriented statements need more validation and implementation. LA-V4 will top the priority list: It is a vulture-derived isolate of *Ligilactobacillus* with desirable strain-level characteristics in vitro, and with a well-controlled in vivo efficacy and safety call will be made before any application-dependent inferences can be made. Because many vulture species are of conservation concern, the present work relied on non-invasive fecal sampling and aims to support microbiome-based discovery without additional disturbance to wildlife

## Figures and Tables

**Table 1:** Biochemical test result of isolate LA-V4.

	LA-V4		LA-V4
Lactose	+	Salicin	+
Inulin	+	Mannitol	+
Raffinose	+	Maltose	+
Sucrose	+	Cellobiose	+
Sorbitol	+	Aesculin	+
Sodium hippurate	−		

**Table 2:** Antibiotic drug sensitivity test of the isolates (S = susceptible; I = intermediate; R = resistant).

	Antibiotic	LA-V4
Aminoglycoside	Gentamicin	S
	Streptomycin	S
	Kanamycin	R
β-lactam	Cefoperazone	S
	Amoxicillin	S
	Penicillin	S
	Oxacillin	I
	Cefazolin	S
Tetracycline	Doxycycline	S
	Tigecycline	S
Quinolone	Levofloxacin	S
	Ciprofloxacin	I
Macrolide	Erythromycin	R
	Tilmicosin	R
Chloramphenicol	Chloramphenicol	S
	Florfenicol	S
Oxazolidinone	Linezolid	S
Lincosamide	Clindamycin	S

## Data Availability

All data supporting the findings of this study are included within the article.
